# Anti-inflammatory role of radiation therapy for pilot treatment of refractory erosive pustular dermatosis of the scalp

**DOI:** 10.1016/j.jdcr.2026.04.064

**Published:** 2026-05-08

**Authors:** Benazir Merchant, Julian Henke, Caroline Serafini, Myer Khan, Zakaria Khan, Hadequa Noor Khan, Travis Blalock, Jamie MacKelfresh, Mohammad K. Khan

**Affiliations:** a Medical College of Georgia, Augusta, Georgia; b Department of Radiation Oncology, Emory University School of Medicine, Atlanta, Georgia; c Georgia Institute of Technology, Atlanta, Georgia; d University of Washington, Seattle, Washington; e Karachi Medical and Dental College, Karachi, Pakistan; f Department of Dermatology, Emory University School of Medicine, Atlanta, Georgia

**Keywords:** clinical research, erosive pustular dermatosis of the scalp, general dermatology, lower-dose radiation therapy, radiation oncology

Erosive pustular dermatosis of the scalp (EPDS) is characterized by large erythematous, erosive, crusted plaques on atrophic skin that progress to scarring alopecia. EPDS can mimic squamous cell carcinoma (SCC), Brunsting-Perry-type cicatricial pemphigoid, or folliculitis decalvans.^1^^,^^2^ The condition typically affects the elderly, has multiple causes, and exhibits nonspecific clinical and histopathological findings.^1^^,^^3^ Some treatment options include topical corticosteroids and calcineurin inhibitors, systemic steroids and retinoids, or photodynamic therapy.^3^ This report highlights a potential long-term curative approach for managing EPDS. In this investigational pilot, we treated 3 patients with refractory EPDS with varying degrees of radiation therapy (RT) (Table I). RT was recommended following multidisciplinary discussion and failure of prior treatments. Computed tomography–guided RT with en-face electron technique was initiated at variable doses for refractory EPDS.

**Table I:** Patient characteristics with refractory erosive pustular dermatosis

Patient	Age (y)	Gender	EPDS duration	RT dose (Gy)	Fractions
1	49	Female	8 y	24	12
2	78	Male	Progressive (exact duration not specified)	55	20
3	80	Female	3 y	36	6

*EPDS*, Erosive pustular dermatosis of the scalp; *RT*, radiation therapy.

Patient 1 was a 49-year-old female who presented with persistent scalp inflammation with overlapping features of EPDS, lichen planopilaris, and folliculitis decalvans despite multiple treatments including vinegar soaks, mupirocin, high potency topical steroids, doxycycline, methotrexate, and prednisone. The patient was unable to tolerate topical and systemic therapies and declined continuation of pharmacologic therapy. Skin biopsy results were nonspecific and demonstrated a dermal scar. Direct and indirect immunofluorescence testing of skin and serum and enzyme-linked immunosorbent assay were negative for evidence of autoimmune bullous disease. Off-label RT was initiated with a total dose of 24 Gy over 12 fractions daily using subfollicular ablative dosing to alter the scalp microenvironment with marked reduction in symptoms and scale crust. She has remained stable since 2017, with no recurrence of scalp inflammation (Fig 1). Patient 2 was a 78-year-old male with extensive actinic damage in the area of male pattern alopecia with multiple SCCs of the scalp who presented with progressive thick crusts on the scalp. Two scalp biopsies were obtained: 1 showed epidermal ulceration with inflammation and granulation tissue, consistent with EPDS, while the other, from a noncontiguous site, demonstrated SCC in situ. Although the EPDS showed partial response to clobetasol ointment, RT was initiated to treat both the EPDS and SCC in situ, delivering 55 Gy over 20 fractions daily. At 1-year follow-up, the patient declined an in-person visit due to no recurrence. Patient 3 was an 80-year-old female with a 3-year history of progressive ulceration and crusting diagnosed as EPDS after negative bacterial and fungal cultures, multiple biopsies with nonspecific findings, and negative direct immunofluorescence testing. EPDS failed to show significant improvement despite treatments including topical tacrolimus, clindamycin, salicylic acid shampoo, vinegar soaks, dapsone, methotrexate, azithromycin, prednisone, acitretin, and dupilumab. A skin biopsy in an area of persistent tenderness showed SCC. RT was initiated, delivering 36 Gy over 6 twice-weekly fractions, targeting cancer with expanded volume to include the SCC as well as the surrounding EPDS. Eleven months post-treatment, the patient reported notable clinical improvement in the EDPS as well as resolution of her SCC (Fig 2).

**Fig 1:**
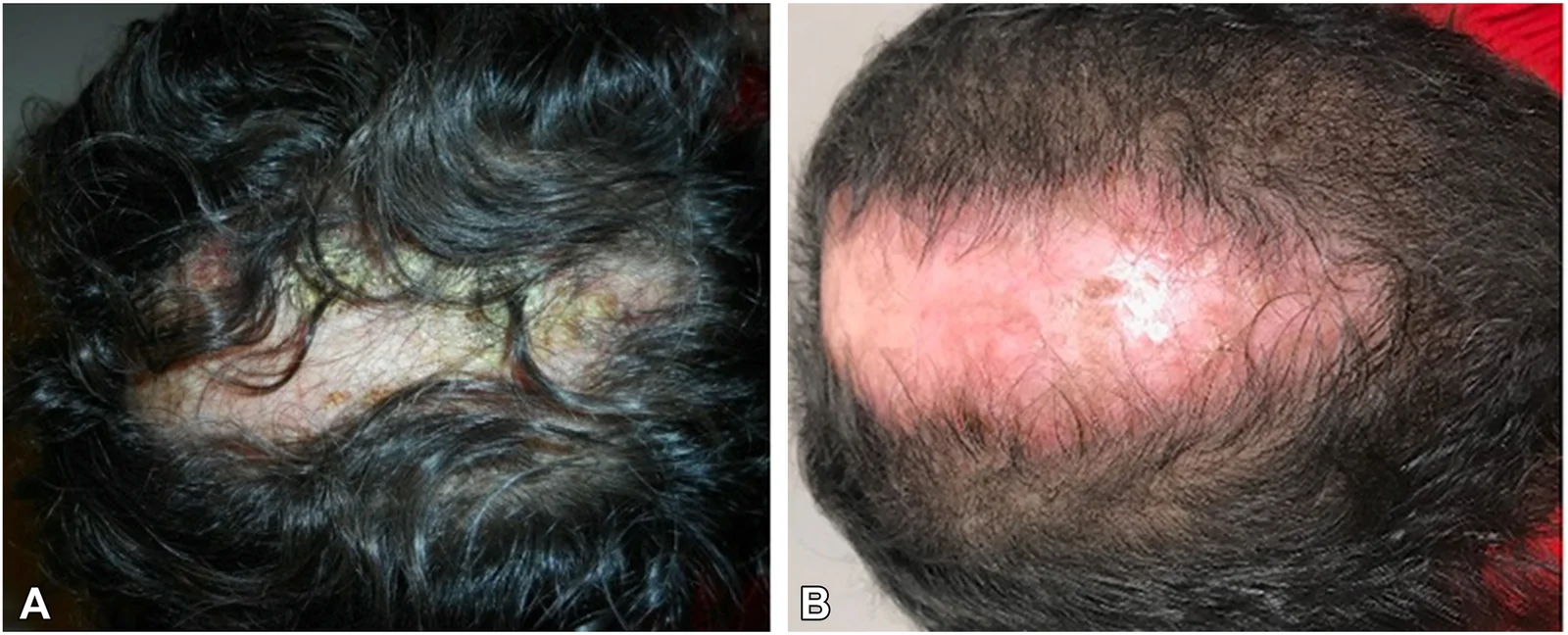
Patient 1: Refractory erosive pustular dermatosis treated with lower-dose RT 24 Gy in 12 fractions at 7-year follow-up. **A,** Refractory erosive pustular dermatosis of the scalp (EPDS) and cicatricial alopecia at initial presentation. **B,** Stable cicatricial alopecia 5 months after completion of radiation therapy (24 Gy in 12 fractions), with no recurrence of EPDS. *RT*, Radiation therapy.

**Fig 2:**
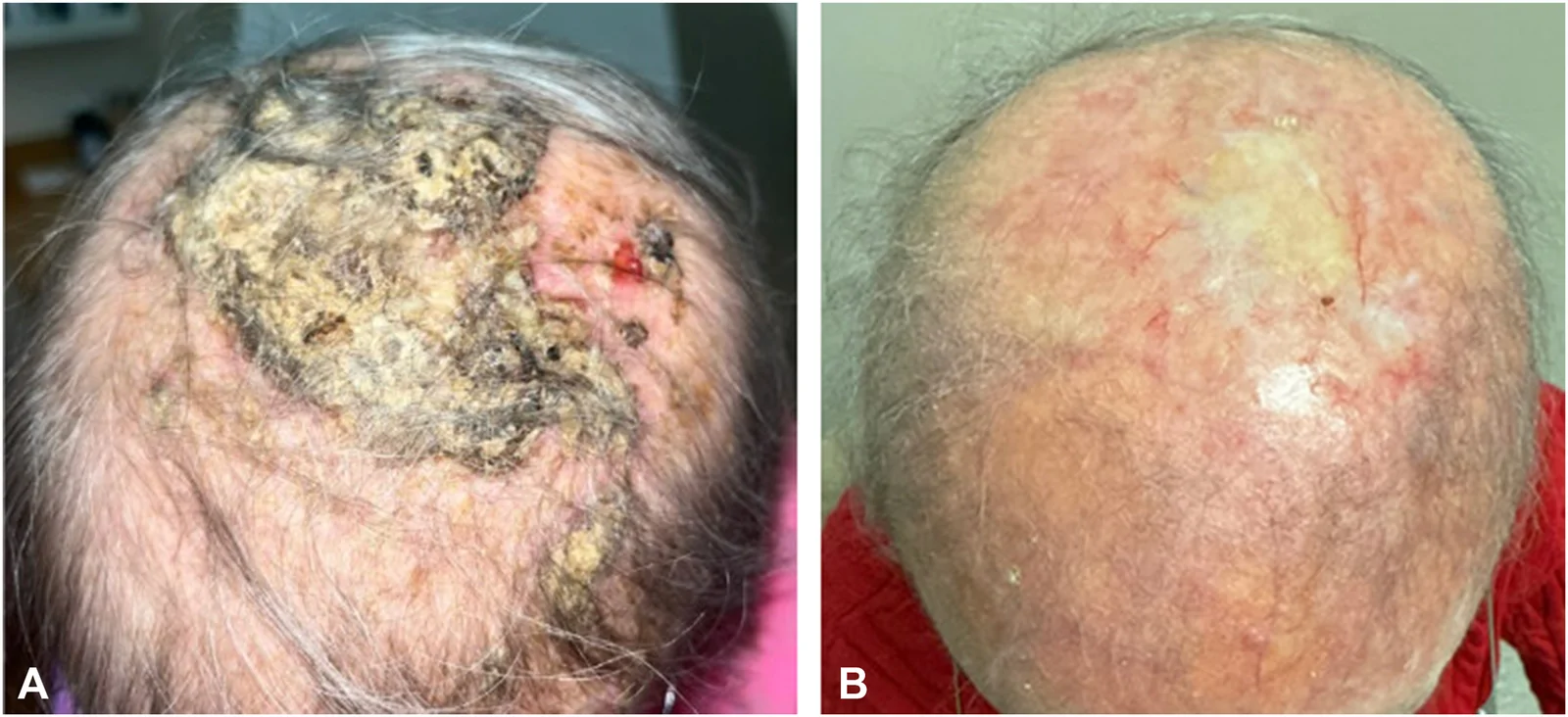
Patient 3: Refractory erosive pustular dermatosis treated with lower-dose RT 36 Gy in 6 fractions at 11-month follow-up. **A,** Refractory erosive pustular dermatosis of the scalp (EPDS) at initial presentation. **B,** Completion of radiation therapy 11-month follow-up visit (36 Gy in 6 fractions), with improvement in EPDS. *RT*, Radiation therapy.

While EPDS has been reported post RT,^3^ our pilot suggests lower-dose RT may actually reduce inflammation. Patients 2 and 3 presented with concomitant EPDS and SCC. Given the dual pathology, higher doses of RT were selected to target both the malignant lesion and the surrounding inflammatory EPDS. Patient 1 had no SCC and thus received off-label lower-dose RT for EPDS. Notably, the flare did not recur following treatment for all 3 patients, suggesting a potential long-term therapeutic role of RT for refractory EPDS, even at higher doses of RT that would destroy SCC. RT may reduce inflammation either via direct cytotoxic effect on overactive inflammatory cells or via macrophage polarization. Dose-dependent RT stimulates macrophage polarization to the M1 or M2 phenotype. The duality of this treatment enables M2 phenotypical cells to exhibit anti-inflammatory effects, while M1 phenotype cells kill tumor cells. This parallels its prior immunomodulatory use in COVID-19.^4^^,^^5^ Larger studies are needed to determine optimal RT dosing, including lower-dose efficacy as in patient 1. If EPDS is primarily impacted by anti-inflammatory effects, then much lower doses could be a key finding in future studies.

A limitation of RT in the management of EPDS is its potential to induce an inflammatory cascade through the generation of reactive oxygen species and proinflammatory cytokines.^6^ Given that EPDS is primarily a neutrophilic folliculitis precipitated by trauma on an actinically damaged scalp, RT may theoretically exacerbate the condition.^7^ Additionally, therapeutic RT is associated with an increased risk of secondary skin malignancies within previously irradiated fields. Future treatment with RT could be limited as therapeutic reirradiation is usually not recommended due to higher complication rates; however, it could be an option for small, localized, or inoperable recurrences after RT, including in elderly patients.^8^

## Conflicts of interest

None disclosed.
